# Delayed intracranial hemorrhage in elderly anticoagulated patients sustaining a minor fall

**DOI:** 10.1186/s12873-018-0179-0

**Published:** 2018-08-24

**Authors:** Nolan Mann, Kellen Welch, Andrew Martin, Michael Subichin, Katherine Wietecha, Lauren E. Birmingham, Tiffany D. Marchand, Richard L. George

**Affiliations:** 1 Summa Health System- Department of Surgery, Akron Campus, Akron, OH USA; 2 Summa Health System- Department of Surgery, Division of Trauma Akron Campus, Akron Ohio, USA Northeast Ohio Medical University, Rootstown, OH USA

**Keywords:** Intracranial hemorrhage, Head injury, Elderly, Fall, Delayed intracranial hemorrhage

## Abstract

**Background:**

Falls are a common cause of hospitalization, morbidity, and mortality among the elderly in the United States. Evidence-based imaging recommendations for evaluation of delayed intracranial hemorrhage (DICH) are not generally agreed upon. The purpose of this project was to evaluate the incidence of DICH detected by head computer tomography (CT) among an elderly population on pre-injury anticoagulant or antiplatelet (ACAP) therapy.

**Methods:**

Data from a Level 1 Trauma Center trauma registry was used to assess the incidence of DICH in an elderly population of patients (≥65 years) who sustained a minor fall while on pre-injury ACAP medications. Counts and percentages are reported.

**Results:**

Data on 1076 elderly trauma patients were downloaded, of which 838 sustained a minor fall and 513 were found to be using a pre-injury ACAP medication. One patient (0.46%) with a DICH was identified out of 218 patients who received a routine repeat head CT. Aspirin and warfarin were the most common pre-injury ACAP medications and 19.27% (42/218) of patients were found to be using multiple ACAP medications.

**Conclusions:**

Universal screening protocols promote immediate-term patient safety, but do so at a great expense with respect to health expenditures and increased radiation exposure. This analysis highlights the need for an effective risk assessment tool for DICH that would reduce the burden of unnecessary screenings while still identifying life-threatening intracranial hemorrhages in affected patients.

## Background

Falls are recognized as one of the primary causes of injury and death in the elderly population [1]. In 2015, 6359 per 100,000 elderly adults (aged 65 and older) experienced an unintentional nonfatal fall, costing the U.S. health care system approximately $31 billion [2, 3]. Intracranial injury as a result of falling is a concern in any patient that falls, but is of principal concern in the elderly population, and even more so among those who use anticoagulant and antiplatelet (ACAP) medications. Individuals taking pre-injury ACAP medications have a two-fold increase in the risk of intracranial injury after blunt head injury [4–6]. Furthermore, elderly adults are the most common age cohort prescribed ACAP medications, making them more susceptible to this risk [6].

Rates of immediate traumatic intracranial hemorrhage following a traumatic injury identified by head CT scan vary in the literature, ranging from 5.1–29.1% [7–13]. In the event that an immediate intracranial hemorrhage has been ruled out, delayed intracranial hemorrhage (DICH) among injured elderly patients on ACAP therapy still remains a concern [14, 15]. Prior studies have concluded that rates of DICH in elderly injured patients on pre-injury ACAP medications are low, ranging from 0 to 6% [8, 9, 13, 16–19]. Most of these analyses have considered limited panels of ACAP medications and have not addressed patients taking multiple ACAP medications. Even in cases where a DICH is detected, neurosurgical operative intervention has been shown to be infrequent [17, 19, 20].

Performing a repeat head computed tomography (CT) after an initial negative head CT is one strategy to evaluate delayed intracranial hemorrhage. Evidence has shown that most DICHs occur within 6–24 h of injury [21, 22], although a recent large multicenter prospective study found that delayed hemorrhages occurred up to 5 days after injury [19]. There is no clear consensus on whether or not to routinely perform repeat head CTs in elderly patients with minor injuries on ACAP therapies, although this is the standard practice in many European trauma centers [23]. Low rates of DICH and even lower rates of subsequent neurosurgical operative intervention have caused many to question the value of the routine repeat head CT [19]. Yet, others still see it as a valuable tool to diagnose DICH, especially since there are no known predictors of DICH [18, 24].

The primary aim of this analysis was to quantify the frequency of DICH in an elderly cohort presenting following a minor fall while taking pre-injury ACAP medications. Prior studies have typically included only specific ACAP medications—most commonly warfarin and clopidogrel. This analysis includes a multitude of ACAP medications which is more reflective of our population. Additionally, this analysis used patients who fell and were admitted to a trauma service as the primary inclusion criteria, rather than discharge diagnosis codes that are assigned retrospectively to select patients for inclusion. This more closely resembles prospective clinical decision-making.

## Methods

This was a retrospective analysis utilizing data from the trauma registry of a Midwestern College of Surgeons verified Level 1 Trauma Center and electronic medical record system. Head CT utilization, results, and medication data were abstracted from the medical record, while all other data elements came from the trauma registry. Data from January 2014–December 2015 was utilized. The present study is not regulated by our institution’s Institutional Review Board as it is not considered human subjects research per the federal definition. Our institutional office of research administration has deemed and approved this to be a quality improvement project and approved the publication of the results.

**Fig. 1:**
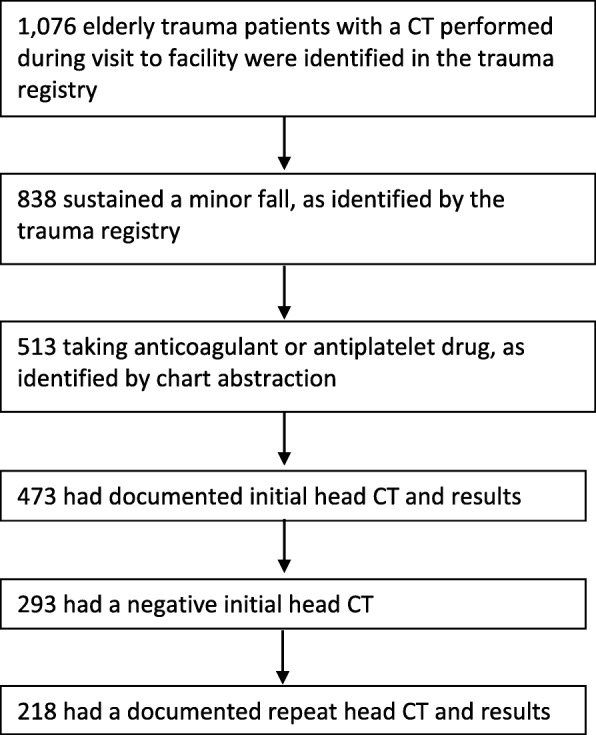
Patient Flow Diagram

Inclusion criteria comprised of being 65 years or older, admission to the trauma service, sustaining a minor fall as defined by the National Trauma Data Standard (NTDS) as a fall from less than 10 ft [25], having a documented head CT scan in the trauma registry, actively taking an anticoagulant or antiplatelet medication at the time of presentation to the emergency room, and having both an initial and routine repeat head CT performed prior to discharge. In order to be admitted to the trauma service at the institution where this analysis was performed evidence of cranio-facial trauma must be present. Many prior studies have relied on discharge diagnoses to limit the analysis to only those patients with documented head injuries whereas the present analysis is limited to those patients who had evidence of cranio-facial trauma without relying on subsequent administrative data. While the populations are very similar, the current analysis does not rely on discharge diagnoses to define the population, but rather, clinical observation that occurred during the trauma-related visit. A wide variety of ACAP therapies included were: warfarin (Coumadin and Marevan), dabigatran (Pradaxa), apixaban (Eliquis), rivaroxaban (Xarelto), dipyridamole (Aggrenox), Enoxaparin (lovenox), heparin, aspirin, clopidogrel (Plavix), prasugrel (Effient), ticlopidine (Ticlid), and ticagrelor (Brilinta). Figure 1 demonstrates how patients were identified for the analysis.

Several demographic variables were exported from the trauma registry including age, sex, race, hospital length of stay, presence of alcohol, injury severity score (ISS), and the initial Glasgow Coma Score (GCS). Data was analyzed using SAS 9.3 (Cary, N.C.).

The primary outcome was the prevalence of DICH identified on repeat head CT scan. DICH was defined as the presence of subdural hematoma, epidural hematoma, subarachnoid hemorrhage, parenchymal contusion, or intraventricular hemorrhage evidenced on repeat head CT after an initial negative head CT. Secondary outcomes included neurosurgical interventions and mortality for patients with DICH and adherence to the departmental practice guideline. The departmental practice guideline is that a routine repeat head CT should be performed for any patient 65 years of age or older with a negative initial head CT who presents within 6 h of a minor fall, while on a pre-injury ACAP medication.

## Results

Trauma registry data was exported on 1076 patients admitted to the trauma service aged 65 years or older during the study period with at least one head CT. Eight hundred thirty-eight (838) were identified as minor fall patients and 513 were found to be taking pre-injury ACAP medications. Figure 1 presents the patient flow diagram. The average age of the sample population was 81.3 years of age. Most cases had injury severity classified as minor (80.28%) or moderate (15.60%). The vast majority of GCS scores fell into the lowest-risk range, which can still be indicative of mild traumatic brain injury. Demographics are presented in Table 1.

**Table 1:** Demographics of Elderly Minor Fall Patients Admitted to the Trauma Service on Pre-injury ACAP Therapy who Received a RRHCT (*n* = 218)

Characteristic	Mean (SD)
Age	81.6 (7.65)
	Mean (IQR)
Length of stay, days	2 (1–4)
	n (%)
Sex	
Female	127 (58.26%)
Male	91 (41.74%)
Race	
Black	9 (4.13%)
White	206 (94.50%)
Other	3 (1.37%)
ISS Group	
0–9 (Minor)	175 (80.28%)
10–14 (Moderate)	34 (15.60%)
15–24 (Severe)	8 (3.66%)
≥ 25 (Very Severe)	0 (0.00%)
Not documented	1 (0.46%)
Alcohol Evident	
Negative	144 (66.05%)
Trace	63 (28.90%)
Yes	9 (4.13%)
Not documented	2 (0.92%)
Glasgow Coma Scale	
3–8	5 (2.30%)
9–12	6 (2.75%)
13–15	202 (92.65%)
Not documented	5 (2.30%)

Table 2 summarizes ACAP medication utilization. The most commonly used ACAP medication was aspirin (54.13%), followed by warfarin (33.03%) and clopidogrel (Plavix) (16.51%).

**Table 2:** Anticoagulation and Antiplatelet Pre-injury Use (*n* = 218)

ACAP Therapy	n (%)
Warfarin (Coumadin and Marevan)	
Yes	72 (33.03%)
No	146 (66.97%)
Dabigatran (Pradaxa)	
Yes	6 (2.75%)
No	212 (97.25%)
Apixaban (Eliquis)	
Yes	11 (5.05%)
No	207 (94.95%)
Rivaroxaban (Xarelto)	
Yes	13 (5.96%)
No	205 (94.04%)
Dipyridamole (Aggrenox)	
Yes	4 (1.83%)
No	214 (98.17%)
Enoxaparin (lovenox)	
Yes	2 (0.92%)
No	216 (99.08%)
Heparin	
Yes	0 (0.00%)
No	218 (100.00%)
Ticagrelor (Brilinta)	
Yes	0 (0.00%)
No	218 (100.00%)
Aspirin	
Yes	118 (54.13%)
No	100 (45.87%)
Clopidogrel (Plavix)	
Yes	36 (16.51%)
No	182 (83.49%)
Prasugrel (Effient)	
Yes	0 (0.00%)
No	218 (100.00%)
Ticlopidine (Ticlid)	
Yes	0 (0.00%)
No	218 (100.00%)

Approximately one-fifth (19.27%, 42/218) of patients presented to the emergency department (ED) on more than one pre-injury ACAP medication. Aspirin and clopidogrel (Plavix) was the most common combination, followed by aspirin and warfarin (Coumadin or Marevan) (Table 3). Out of all ACAP medications, aspirin was most commonly used in combination with another medication, followed by warfarin (Coumadin or Marevan). Two patients presented to the ED on three separate ACAP medications.

**Table 3:** Number of Patients Taking Multiple Pre-injury ACAP Therapies

ACAP Therapy	N
Aspirin + clopidogrel (Plavix)	16
Warfarin (Coumadin or Marevan) + aspirin	14
Apixaban (Eliquis) + aspirin	2
Dabigatran (Pradaxa) + aspirin	2
Rivaroxaban (Xarelto) + clopidogrel (Plavix)	2
Warfarin (Coumadin or Marevan) + Enoxaparin (lovenox)	2
Warfarin (Coumadin or Marevan) + clopidogrel (Plavix)	1
Warfarin (Coumadin or Marevan) + aspirin + clopidogrel (Plavix)	1
Dipyridamole (Aggrenox) + clopidogrel (Plavix) + aspirin	1
Dipyridamole (Aggrenox) + aspirin	1

Out of 513 eligible patients for an initial head CT, 473 (92.22%) received an initial head CT. Two-hundred ninety-three (293) patients had negative initial head CT (61.95%). Of the 293 patients eligible for a routine repeat head CT, 218 were documented with results (74.40%). Of the 293 eligible for a routine repeat head CT, 24 were documented as not ordered because the patient arrived more than 6 h after being injured, 2 were not ordered because the patient expired, and 49 were not ordered for an unspecified reason. One positive routine repeat head CT was identified (0.46%) out of 218. The single patient with a positive repeat head CT survived to discharge without neurosurgical intervention or significant morbidity.

## Discussion

This study identified one patient with a DICH out of 218 patients who received routine repeat head CTs. This patient required no neurosurgical intervention and no change in clinical management. The patient had an ISS of 22 and was taking apixaban (Eliquis) prior to injury. No additional risk factors were identified in this patient.

The lack of surgical intervention in the identified patient is consistent with prior studies that have reported very low levels of surgical intervention in this patient population [8, 19, 26]. Additionally, this analysis identified that 49 patients did not receive a routine repeat head CT. These patients were analyzed further, and it was determined that approximately half of the patients arrived more than 6 h after their injury, thus should not have received a routine repeat head CT, per the departmental guideline. Given that some head CTs were still missed, a larger quality improvement project was conducted to reduce practice variation with respect to repeat head CT ordering in this population.

The literature demonstrates variation in rates of DICH among elderly injured populations (0.3% versus results up to 6%) [8, 19]. One reason for this may be differences in inclusion criteria that can potentially vary (e.g., injury type, age, etc.). Some states, including the state where this analysis was conducted, have specific geriatric trauma triage guidelines established to help protect elderly patients from undertriage which may have the effect of increasing the denominator for elderly patients screened for DICH [27]. Undertriage occurs when patients are taken to facilities that do not have the resources required to treat their acute health problems, and can lead to poor patient outcomes and poor resource utilization [27, 28]. Elderly populations have been found to be at greater risk of undertriage which is why some states have moved to triage models where age is a criteria for referral to a level I trauma center and trauma activation [28]. Furthermore, it is possible that rates of DICH diagnosed by CT have decreased due to increased overall usage of CT scans of the head in minor trauma (increasing the denominator to include more minor patients).

There continues to be significant debate in the literature regarding best clinical practices with respect to screening for DICH in elderly patients with minor injuries on ACAP therapies. Overutilization of CT scans is undesirable from a financial perspective and raises concerns over radiation exposure, although this concern is somewhat reduced in the elderly population. While increased CT utilization has drawbacks, DICH in patients taking anticoagulant agents has historically been reported as significant contributor to both morbidity and mortality [29] however a recent large multicenter study has suggested that this risk is less pervasive [19]. In response to these reports, a 2002 initiative by the European Federation of Neurological Societies (EFNS) recommended a 24-h observation period followed by a repeat head CT for all anticoagulated patients with minor head trauma [23]. An Italian study by Menditto investigated the EFNS recommendations and reported a 6% rate of DICH in patients receiving warfarin [8]. As a result, many trauma centers in Europe have offered admission and repeat head CT to all anticoagulated patients with head trauma. This gradually became the standard at many institutions worldwide, and is the model for the practice guideline at the institution where this work was conducted.

Our findings of DICH are consistent with previous studies and highlight the need for a more efficient way to detect DICH. Current protocols using repeat CT imaging are resource intensive. One study found that the average cost to detect a single DICH was $1,016,960 when using a universal screening protocol, which has caused some to question the cost-effectiveness of this method of screening [30]. For this reason, future research into risk factors that can accurately predict DICH is imperative.

Some improvements have already taken place that have likely decreased rates of DICH. The quality, efficiency, and speed of CT imaging has improved significantly overtime. This improvement has allowed for decreased radiologic artifact, decreased motion artifact, increased number of images, and increased quality of images all in an abbreviated timeframe [31]. This allows for a more confident exclusion of pathology with CT imaging. Thus, it is possible that some previously reported DICHs were simply missed immediate intracranial hemorrhages. Furthermore, it is possible that service delivery innovations, such as geriatric consultations for geriatric trauma patients, could also improve processes and subsequent outcomes for geriatric patients [32].

Improvements in the management of patients on blood thinners may also contribute to a decrease in the overall number of DICH detected. Known risks of ACAP agents have led to a significant improvement in anticoagulant management. This has included the development of carefully monitored warfarin clinics, public awareness regarding blood thinners, and the development of new and safer anticoagulant agents. Several direct oral anticoagulants (DOACs) have been introduced (apixaban, rivaroxaban, and dabigatran). The safety of these DOACs in the trauma population has recently been assessed. DOACs have a considerably lower rate of the development of intracranial hemorrhage compared to warfarin (24% vs. 31%) [33]. While there has likely been benefit, the DOACs have few established protocols for reversal of anticoagulation, although work continues to be done in this area [33–36]. Research has shown that utilization of multiple ACAP medications is associated with increased bleeding risk [37]. The present analysis, with approximately 20% of elderly patients were utilizing more than one ACAP medication, double the rate of the recent Chenoweth et al. [19] study, still does not suggest that dual or triple pre-injury ACAP use is associated with increased risk of DICH, although our numbers were relatively small.

The volume of CT scans has increased due to the wide availability of timely CT imaging, which may have the effect of increasing the probability of detecting DICHs. In 2007, there were an estimated 75 million CT scans performed [38]. Since then, the number of CT scans performed has increased by approximately 10% per year [39]. Increased screening will likely lead to increased detection. An unintended consequence of the increase in CT utilization as a result of the EFNS repeat head CT guideline is that this increases length of stay, and as such, may result in a greater consumption of health care resources from the trauma service, or other hospital service lines which subsequently increases costs for patients. Given these considerations, a comprehensive examination of the costs and benefits associated with this screening protocol is warranted.

This analysis was not without limitations. As a retrospective analysis, the project was subject to some intrinsic limitations including the potential for missed enrollment due to possible errors in documentation and varying adherence to our institutional practice recommendations which could have resulted in selection bias. In a separate analysis for an associated lean six sigma project, factors associated with missing a RRHCT including age, gender, GCS, injury severity, aspirin-only, or warfarin utilization were examined. No factor was statistically significantly associated with missing a RRHCT in logistic regression analysis. Furthermore, in discussions with providers about why they believed RRHCT were missed, the most common responses were (1) not being aware of the departmental guideline, and (2) forgetting to adhere to the departmental guideline. For these reasons, the risk of selection bias is somewhat reduced since the evidence suggests that missing RRHCTs were missing at random. Given our usage of a trauma registry that is maintained by trained trauma registrars, the possibility of documentation errors is also somewhat reduced. Adherence to institutional practice recommendations, namely performing the repeat head CT, was the center of a larger quality improvement project aimed at reducing variation in clinical practice with respect to this guideline. The project has led to significant improvements in compliance, thus in the future it would be possible to reassess our rate of DICH with less potential for selection bias. Given the very low incidence of DICH at our institution and others, there was an inability to power and match our study to examine individual pharmacologic agents. The state in which the analysis was conducted has specific geriatric trauma triage criteria which may differ from other states, potentially limiting generalizability to trauma centers without such geriatric-specific trauma triage criteria [26]. Lastly, long-term outcomes were outside the scope of this analysis, and as such we were unable to examine long-term complications and outcomes.

A strength of this analysis is that there was greater inclusion of ACAP medications than previous studies. This project included DOACs and aspirin which many prior analyses have not included. Furthermore, this study presents utilization rates for multiple ACAP medications which is a unique contribution to the literature.

## Conclusion

The incidence of DICH in geriatric patients using a broad range of ACAP therapies prior to a minor fall was very low. The rate of surgical intervention following a DICH was even lower. Some have recommended against universal DICH screening protocols, but without evidence-based guidelines that effectively stratify DICH risk, this recommendation is somewhat risky, even if the risk is a low probability event. Future research should investigate a broad spectrum of ACAP medications and work to identify risk factors for DICH so that patient safety and clinical quality are not sacrificed for operational efficiency.

## Abbreviations

ACAP: Anticoagulant or antiplatelet
CT: Computed tomography
DICH: Delayed intracranial hemorrhage
DOAC: Direct oral anticoagulant
EFNS: European Federation of Neurological Societies
NTDS: National Trauma Data Standard
